# Young adults not in education, employment, or training (NEET): a global scoping review

**DOI:** 10.1186/s12889-025-24781-y

**Published:** 2025-10-08

**Authors:** Mari Gunnes, Kristin Thaulow, Silje L. Kaspersen, Chris Jensen, Solveig Osborg Ose

**Affiliations:** 1 SINTEF Digital, Health Services Research, Trondheim, Norway; 2 Norwegian National Advisory Unit on Occupational Rehabilitation, Rauland, Norway

**Keywords:** NEET, Youth unemployment, Mental health, Social exclusion, Education, Labour market

## Abstract

**Background:**

Young adults not in education, employment, or training (NEET) represent a significant global issue, with varying challenges across different countries. Research indicates a strong association between NEET status and negative outcomes such as mental health difficulties, low self-esteem, and social exclusion, though the direction of causality is often complex and bidirectional. This scoping review aimed to provide a comprehensive mapping of international research on NEETs, including risk factors, characteristics, and effective interventions to inform future policy and practice.

**Methods:**

This scoping review was conducted following the Joanna Briggs Institute (JBI) framework, incorporating the PRISMA-ScR checklist. The review included six key stages: identifying the research question, identifying relevant studies, selecting studies, charting the data, collating, Summarizing, and reporting the results, and consultation. A systematic search was conducted in PubMed, Scopus, and Web of Science, covering literature from 2021 to April 2024. Eligibility criteria were established using the population-concept-context (PCC) framework.

**Results:**

A total of 159 studies were included, classified into 11 topics. The review identified a diverse range of factors that influence the status of NEET, including individual, family, and systemic elements. Key determinants such as cognitive abilities, noncognitive skills, and socioeconomic background were highlighted. Psychological issues, including mental health problems and low self-esteem, were prevalent among NEETs. Social issues such as inequality, discrimination, and social exclusion were also significant. The review found that NEET status is associated with long-term socioeconomic disadvantages, including lower educational attainment, higher unemployment rates, and increased mental health risks. The effectiveness of the interventions varied, some showing positive outcomes in terms of employment and mental health, while others had limited impact.

**Conclusions:**

The NEET phenomenon is complex and requires a holistic approach that integrates the health, welfare and work life sectors. Effective interventions should be tailored to the specific needs of NEET individuals, considering their mental health, self-esteem, and social connections. Policymakers should focus on developing comprehensive support systems that address the diverse challenges faced by NEETs, ensuring sustainable transitions to education, employment, or training. More research is needed to explore the long-term effectiveness of various interventions and to identify best practices to support NEETs worldwide.

**Supplementary Information:**

The online version contains supplementary material available at 10.1186/s12889-025-24781-y.

## Introduction

The concept of young adults not in education, employment, or training (NEET) has emerged as a critical social and economic issue over recent decades. Initially formulated in the United Kingdom due to changes in benefit regimes in the late 1980 s that rendered British school-leavers ineligible for unemployment benefits until they turned 18, the NEET concept soon gained popularity beyond the UK. The term has been adopted in almost all EU member states as well as in countries such as Japan, New Zealand, Taiwan, Hong Kong, and South Korea [1].

**Fig. 1:**
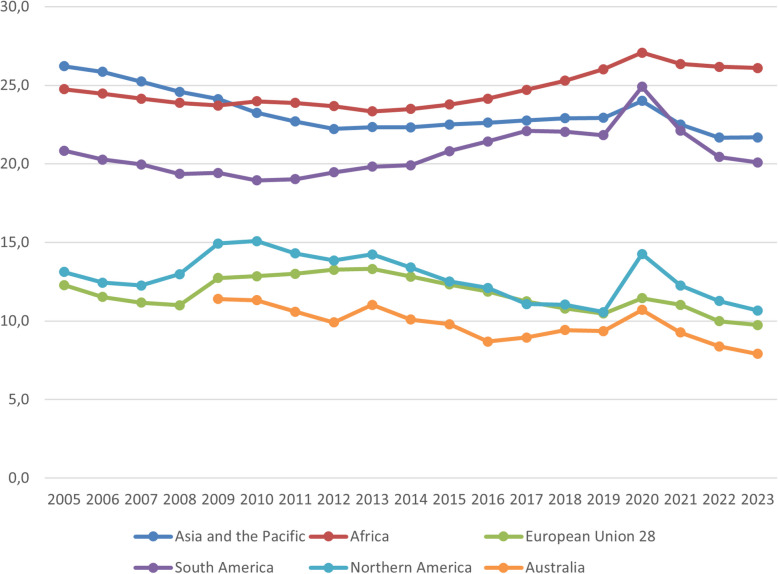
Proportion of youth aged 15-24 years worldwide not in education, employment, or training (NEET) worldwide from 2005 to 2023. Source: SDG indicator 8.6.1, ILOSTAT

Although the NEET concept has gained global relevance [2], much of the existing literature and previous reviews have concentrated on Western and high-income countries, often overlooking low- and middle-income contexts [3] and the growing body of digital interventions [4]. At the same time, the prevalence of NEET status remains a pressing global concern, with significant regional disparities. Figure 1 reveals Substantial variation in NEET rates across regions from 2005 to 2023. Africa and Asia and the Pacific consistently report the highest NEET rates, both hovering around 25% throughout the period, with Africa showing a noticeable increase between 2015 and 2019. South America follows a similar trajectory, with a moderate increase leading up to a sharp peak in 2020, likely related to the COVID-19 pandemic, before declining again. In contrast, North America and the European Union (EU-28) exhibit lower rates, with the EU showing a Steady decline from 13% in 2005 to below 10% in 2023. Australia consistently reports the lowest NEET rates, reaching a low of approximately 8% in 2023. Across nearly all regions, 2020 marks a temporary spike in NEET rates, highlighting the pandemic’s impact on youth engagement.

The concept of youth withdrawal or “stagnant youth” varies in meaning across different cultures, contexts and populations [5]. For instance, *hikikomori*, characterised by the avoidance of social interactions for more than six months, has been recognised as a serious social problem in Japan for over 20 years [6]. Additionally, in 2021 a phenomenon known as the “lying flat movement” rapidly spread throughout China’s social media [7]. In Europe, the NEET concept is well-established, and Holte underscores the importance of understanding NEET status not only through quantitative measures but also through the lived experiences of young individuals [5]. Across the literature, terms such as “disengaged youth,” “youth at risk,” and “inactive youth” are sometimes used interchangeably with NEET. For the purpose of this review, we adopt an inclusive definition of NEET, referring to individuals aged 16-35 who are not engaged in education, employment, or training. While international organisations Such as UNESCO and the ILO typically define NEET as those aged 15−24 or 15-29, the extended age range to 35 is increasingly used in policy and research contexts to reflect prolonged transitions to adulthood, particularly in low- and middle-income countries [8, 9].

The consequences of being NEET are profound and multifaceted, including socio-economic disadvantages, increased risk of mental health issues, and diminished future opportunities. NEET status is linked to lower well-being, increased social exclusion, reduced economic growth, higher suicide risk, criminal behaviour, and unemployment, with higher education levels acting as a mitigating factor [10–12].

The reasons for becoming NEET are complex and multifaceted, involving a range of demographic, familial, educational, socio-economic, and health-related factors [10]. These are further shaped by country-level factors such as existing policies, labour market and welfare regimes, and economic structures [13, 14]. Additionally, within-country variations, including regional differences and the rural-urban divide, highlight disparities in opportunities for young people based on their place of residence [15]. Understanding these risk factors, characteristics, and consequences is crucial for developing targeted interventions and policies.

Approaches to supporting NEETs vary but often include vocational training programmes, educational reintegration technologies, employment services, and community-based support. These interventions recognise that factors like self-esteem, mental health, health values, beliefs and norms, social connections, and life skills play crucial roles in transitioning NEETs into education, employment, or training [16, 17].

The policy landscape surrounding NEETs is diverse, with various countries adopting different strategies to address the problem. Analysing the impact of these policies can provide valuable insights into successful frameworks and highlight gaps. European policymakers, for example, have implemented a wide range of responses to engage young people in employment, training, or education [15].

A holistic approach that integrates health, welfare, and working life sectors addresses the multifaceted nature of the NEET concept and ensures more effective and sustainable solutions, eventually benefiting both individuals and society as a whole. Hence, this scoping review aims to provide a comprehensive overview of the NEET research field by identifying and synthesising evidence on the characteristics, risk factors, and consequences of NEET status; evaluating the range of interventions supporting NEETs; and drawing implications for policy and practice.. This review expands on prior reviews [18] by including a more recent body of literature and placing particular emphasis on non-Western contexts. In doing so, it seeks to provide a more inclusive and policy-relevant evidence base. By synthesising current evidence, the review seeks to inform the development of more effective policies and practices that can better support NEET individuals in their transition to education, employment, or training.

## Method

This scoping review was conducted in accordance with the framework outlined by the Joanna Briggs Institute (JBI) [19] building on the foundational work of Arksey and O’Malley [20] and Levac and colleagues [21]. The JBI approach to conducting and reporting scoping reviews aligns with the Preferred Reporting Items for Systematic Reviews and Meta-Analyses (PRISMA) Extension for Scoping Reviews checklist [22], which was utilised as a guide for reporting the results. The process followed the six key stages: 1) identifying the research question, 2) identifying relevant studies, 3) selecting studies, 4) charting the data, 5) collating, summarizing and reporting the results, and 6) consultation.

### Stage 1: Identifying the research questions

We conducted preliminary literature searches to map the field of NEET research and to obtain an overview of key concepts, terminology, and relevant keywords. Following this initial exploration, we decided that the overarching aim was to provide a comprehensive overview of the NEET research landscape. Through this approach, the scoping review aims to contribute to a deeper understanding of the NEET phenomenon and to inform the development of more effective policies and practises that can better support NEETs in their transition to education, employment or training from a global perspective. In particular, we sought to identify and synthesise the current evidence-based research available across various dimensions, including i) examining the characteristics, risk factors and consequences of being NEET; ii) assess the range of interventions supporting NEETs; and iii) draw implications for policy and practice.

### Stage 2: Identifying relevant studies

Eligibility criteria were established, as shown in Table 1, and search terms were identified using the population-concept-context (PCC) framework provided by JBI [19]. Keywords targeting the population of interest included young adults, teenagers, adolescents, youth, students, juveniles or pupils who are unemployed, “out of school”, “out of work”, or NEET. Conceptual keywords included terms related to return to work, vocational rehabilitation, reintegration, and evidence-based practices. Specific components of these interventions encompassed peer support, group-based activities, counselling, outdoor activity, physical activity, nutrition, art, dance, music, sleep, yoga, lifestyle changes, and circadian rhythm.

**Table 1:** Eligibility criteria

PCC element	Inclusion criteria	Exclusion criteria
Population	Young adults aged 16 to 35 (or aged as defined in study) who are currently outside the workforce or education system.	Focusing exclusively on sub-populations with severe and specific medical conditions (e.g., severe neurological or psychiatric diseases, heavy substance abuse, individuals in need of suicidal treatment).
	No restrictions based on gender, ethnicity, socioeconomic factors, or regional variations.	
Concept	Research of all kinds (methodologically) including the NEET population with the aim of describing the phenomenon or facilitating the process of returning to work or education.	Lack of focus on describing population or facilitating NEETs’ return to work or education.
		Research not about humans.
Context	Research conducted in various geographical and socio-economic settings, including high-, middle-, and low-income countries. Language limited to English. Publication period: January 2021-April 2024.	Studies conducted in contexts not relevant to health, welfare or working life sectors. Articles on the “Individual Placement and Support” (IPS)-model.
		Research on COVID-19 and pandemic related research.

To ensure exclusion of articles beyond the scope of this review, we added keywords related to serious illnesses such as stroke, psychosis, cancer, injury, chronic disease, schizophrenia, as well as “individual placement and support” (IPS), which is a specific intervention developed for individuals with severe illness. Additionally, research specifically targeting COVID-19 and pandemic-related disruptions was excluded, as our aim was to focus on generalisable NEET trends and interventions rather than temporary effects. Although the search period (2021 - 2024) overlaps with the pandemic, it was chosen to capture the most recent and policy-relevant research. Boolean phrases, combinations of keywords, and, where appropriate, filters for publication date and language were employed. The full search Strategy is detailed in Supplementary Material 1.

Three databases, PubMed, Scopus, and Web of Science, were systematically searched by two researchers for relevant literature from 2021 until the search was conducted (April 10, 2024). These databases were selected to cover a broad range of disciplines, including education, health, and psychology.

We included English-written, peer-reviewed original research papers and reports published in academic journals within the last three years (i.e., 2021 to 2024). Non-original research papers (e.g., reviews, editorials, opinion pieces/commentaries, book chapters, conference proceedings, protocols, pre-prints, dissertations, validation/reliability studies) were excluded. This decision was made to focus the review on primary, evidence-based research and avoid duplication of findings already captured in the original studies. Studies were also excluded if they focused solely on subpopulations of younger adults with specific and severe diseases or conditions (e.g., severe neurological or psychiatric diseases, heavy substance abuse, or those in need of suicidal treatment), or if they focused solely on diagnostic-specific interventions to individuals with severe diseases or interventions targeted solely to individuals outside the labour force, such as individuals with severe physical or mental disabilities.

### Stage 3: Selecting studies

Citations from each database were captured in Zotero reference manager and exported to Covidence. The latter is a web-based software platform designed to help researchers collaborate in producing systematic reviews and other knowledge synthesis projects. After excluding duplicates automatically in Covidence, three researchers independently screened the titles and abstracts to exclude irrelevant studies and to identify articles for possible inclusion. Articles selected for possible inclusion were screened in full text, categorised according to emerging topics, and finally included or excluded based on the eligibility criteria. These decisions were guided by the eligibility criteria outlined in Table 1, which served as a reference framework during the screening process. Disparities were resolved through discussion between four of the authors.

### Stage 4: Charting the data

The articles that met the inclusion criteria were initially sorted into broad thematic categories based on emerging topics identified during manual review. These preliminary groupings were later refined and reorganised through discussions among the authors. As a result, the final synthesis was structured around 11 thematic categories, which are reflected in the Results section and in the coding structure used for data extraction (see Supplementary Material 2).

The following information from each article was extracted to an Excel spreadsheet: author(s), title, year of publication, journal, target population, aim, study design, methodological approach (i.e., quantitative, qualitative, mixed-methods), type of data and analysis, country, main results, and conclusions. In addition, specific content related to the main topics, such as outcome assessment, content of intervention, or future recommendations, were extracted.

### Stage 5: Collating, summarising, and reporting the results

The included articles were summarised and presented in evidence tables (Supplementary Material 2). Furthermore, the results were categorised and presented as a narrative summary of the objectives and findings in categories emerging from the included literature. During this stage, we employed AI tools in compliance with the Springer Nature AI Policy to support the collation and reporting of results. The AI PDF Summarizer was used to extract data from some of the articles, with all the extracted information quality-checked and verified by the authors. Also, ChatGPT (version 4) was used to refine and condense the text for clarity and conciseness, with all modifications reviewed and approved by the authors to ensure integrity of the manuscript. To ensure transparency, AI was employed exclusively for summarisation and language refinement; it played no role in interpretive or analytical decisions.

### Stage 6: Consultation

The consultation process was carried out in collaboration with one of the co-authors (C.J.) who is an experienced leader of a Norwegian national competence centre for vocational rehabilitation. Their extensive experience in implementing vocational programmes for youth offered valuable insights into the practical challenges faced by NEETs in transitioning to education or employment. Discussions and collaboration ensured the results, particularly those related to interventions, were grounded in current vocational rehabilitation practices and provided valuable context for the findings.

## Results

**Fig. 2:**
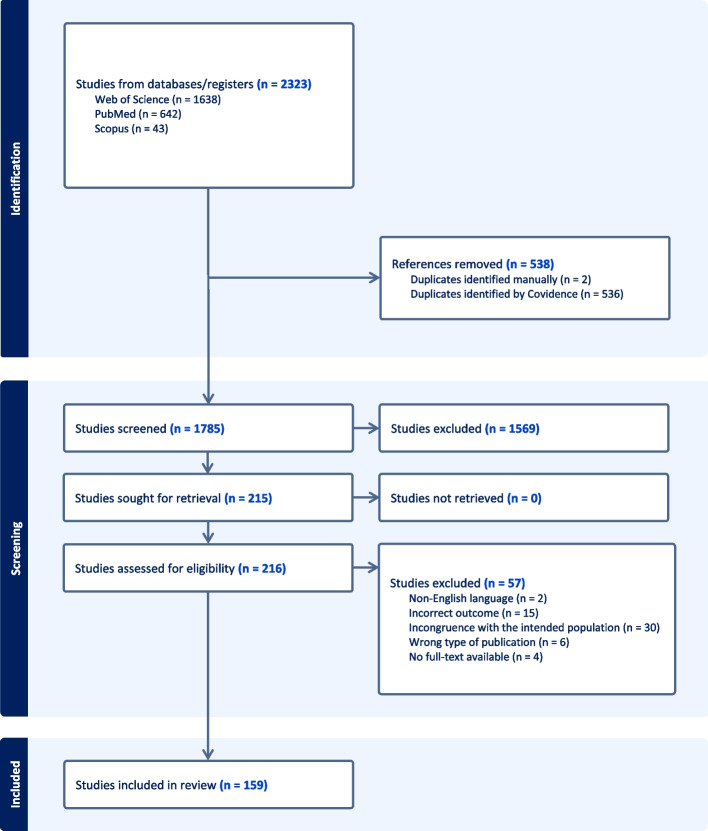
PRISMA flow chart illustrating the study selection process

The search and selection process for this scoping review is summarised in Fig. 2. A systematic search conducted across three databases in mid-April 2024 identified 2,323 citations. After removing duplicates, 1,785 unique citations remained. Titles and abstracts were screened against predefined eligibility criteria, resulting in 216 citations deemed relevant. Full texts of these citations were then obtained and further screened for inclusion. Of these, 57 were excluded for various reasons; incongruence with the intended population ($n=30$), incorrect outcome ($n=15$), wrong type of publication ($n=6$), no full-text available ($n=4$), and non-English language ($n=2$). Finally, a total of 159 studies were included in the present scoping review.

As shown in Table 2, we manually re-coded the 159 articles into 11 different categories to capture the main areas of focus within NEET-related research. This categorisation reveals a strong focus on interventions (26 percent of studies), followed by detailed descriptions of the NEET population and labour market issues.

**Table 2:** Number of articles and topics. The numbers in parentheses (1–11) refer to the 11 thematic categories used to synthesise the included studies, as referenced throughout the Results section

	Number	Percent
Description of NEET:		
Characteristics of NEET (1)	23	14
Psycological issues (2)	24	15
Social issues (3)	9	6
Childhood adversity (4)	3	2
Labour market topics:		
Cyclical variation (5)	6	4
Cross-country comparisons (6)	12	8
Employing NEET (7)	18	11
Interventions (8)	41	26
Consequences of being NEET (9)	7	4
Perspectives on NEET (10)	10	6
Other topics (11)	6	4
Total	159	100

**Table 3:** Number of articles from each continent

	Number of publications	Percent
Africa	19	12
Asia and the Pacific	21	13
Australia	9	6
Europe	91	57
Northern America	11	7
South America	6	4
More than one continent	2	1
Total	159	100

Most studies were conducted in Europe, with approximately 60 % of the research coming from countries of the European Union and associated European nations, while studies from South America and Australia remained underrepresented, as shown in Table 3. Twenty-six (16 %) of the included studies were from non-Western countries.

The largest category focused on interventions (26 %), including employment support programmes, vocational rehabilitation, and training initiatives. Given its policy-relevance, this category is presented in greater detail. The second largest category, psychological issues (15 %), explored mental health challenges that hinder NEETs’ integration into education or employment. Studies on the characteristics of NEETs (14 %), identified demographic, social, and economic factors influencing NEET status, while social issues (6 %) addressed broader societal factors, such as social exclusion. A small number of studies (2 %) examined how childhood adversity impacts the likelihood of becoming NEET.

Labour market issues, including employing NEETs (11 %), cross-country comparisons (8 %), and cyclical variation (4 %), also formed significant areas of focus. The consequences of being NEET (4 %), were explored in terms of long-term impacts on health, economic stability, and social outcomes. Perspectives of NEET (6 %), and other topics (4 %) were less frequently examined.

The majority of studies were cross-sectional and quantitative in nature, with fewer longitudinal or mixed-methods designs, particularly among studies conducted outside high-income countries. A total of 67 % ($n=106$) of the studies were quantitative, based on Survey data, register data, or international databases Such as Eurostat and the World Bank. Qualitative studies accounted for 24 % ($n=38$), including fieldwork, document analysis, and large-scale interview studies. The remaining 9 % ($n=15$) employed mixed methods, typically combining interviews with survey data.

### Description of NEET

#### Characteristics of NEET

Twenty-three of the studies were categorised within “NEET characteristics” [6, 23–44]. Most were observational studies using quantitative methodology (survey or register data), with only four adopting a qualitative approach [28, 37, 40, 44]. Ten studies identified cognitive ability, non-cognitive skills, and socio-economic factors as key determinants of NEET status [6, 24, 27–29, 34, 39, 41, 42, 44]. Research from Turkey [41] and China [42] highlighted education’s role in mitigating NEET status, while studies from South Africa [44] and Chile [43] emphasised the need for gender-specific approaches.

A Danish study explored the direct and indirect effects of cognitive abilities, non-cognitive skills, and socio-economic background on NEET outcomes [6, 28, 41, 42]. Similar findings were noted in English adolescents using longitudinal data [29]. In Russia, NEET status was found to be heterogeneous, with NEET-inactivity linked to primary or vocational education, and NEET unemployment associated with higher education [39]. Employment hope was also important for outcomes in NEETs [23].

NEET individuals have been found to report poorer health, lower psychological well-being, higher alcohol use and sexual risk behaviours compared to non-NEETs [23, 25–27, 38, 45]. Gender differences were notable, with NEET women often worse off than men [26, 31, 32, 34–36, 43, 44], particularly in Norway [32], Italy [34, 35], and the UK [31]. In South Africa, gender-specific dropout patterns were observed, with males quitting due to poor academic performance and females leaving for family-related reasons [44].

Four studies focused on transitions and employment pathways [26, 33, 40, 41]. An Australian study found that successful employment transitions were linked to improved well-being, with job quality being crucial [26]. A US study analysed the patterns of disconnection and reconnection, emphasising the need for interventions to support youth in reintegration into education and employment pathways [33]. Research from Turkey highlighted the impact of involuntary job loss within the household on youth employment transitions, with females more likely to gain employment after the household head’s job loss [41].

Policy responses, such as the reinforced Youth Guarantee across EU Member States, acknowledge structural vulnerabilities contributing to NEET status [46]. While NEET rates have declined in some countries, certain subgroups, such as women with caregiving responsibilities and the long-term unemployed, remain disproportionately at risk [36]. Studies from Spain and Latvia revealed barriers preventing early school leavers from returning to education, underscoring the need for tailored support and highlighting the uneven effectiveness of broad policy interventions [37, 47].

#### Psychological issues

Research on psychological issues among NEETs has employed both quantitative approaches using large-scale surveys, longitudinal data, and administrative records [48–59], as well as mixed-method approaches [60]. Most studies originated from Northern Europe, including Sweden, the Netherlands, Denmark, Finland, UK, and Norway. Contributions also came from Ukraine, China, the USA/Canada, and Australia. Three of the articles were from South Korea and cover topics like stigma, self-esteem, depressive symptoms [61, 62] and social isolation [63].

Common findings indicate that internalising problems, such as anxiety, depression, low self-esteem, and somatic complaints (e.g., headaches and sleep difficulties), are key risk factors for NEET status [48, 51, 52, 54, 56–59, 64]. These issues negatively impact well-being and hinder adaptation to both educational and work environments.

Externalising behaviours, including aggression, impulsivity, and conduct issues, also impair the ability to engage in school or work environments [48, 57, 58, 65]. For example, a Swedish study highlighted the high risk of labour market marginalisation amoung young adults with ADHD, leading to long-term unemployment, sick leave, and disability pensions [66]. Other research emphasised the interplay between internalising and externalising problems during adolescence and their link to later NEET status [48, 51, 54, 56, 57, 59].

Depressive symptoms, such as persistent sadness, irritability, suicidal thoughts, and sleep disturbances, are consistently associated with increased risk of NEET status [49, 51, 52, 59, 64]. Personality traits, such as emotional instability, sensitivity to criticism, and ineffective task strategies, also exacerbate vocational impairment [50, 67]. Finnish research found that school dropouts were associated with psychiatric and neurodevelopmental disorders, including learning disabilities and autism spectrum disorders, increasing the vulnerability of NEET [53].

Low mental resilience, poor self-confidence, and a lack of coping skills were additional factors contributing to NEET risk [55, 56, 60]. A Ukranian study emphasised the psychological impact of unemployment, highlighting financial stress, social status loss, and uncertainty in mental distress [68]. Another study, found that participants with severe depressive symptoms were more likely to be unemployed for 6 months or more and highlighted a lack of social support compared to those without severe depressive symptoms [69]. Similarly, Chinese research found that low perceived social support and stress increased risk of depression and insomnia, suggesting that emotional regulation interventions could be beneficial [11, 12].

Studies also highlighted the association between low educational levels and higher depressive symptoms among NEETs [49]. Early intervention addressing both educational and psychological needs is essential for reducing long-term NEET rates [51].

#### Social issues

Several studies highlighted social issues, although few focused exclusively on them. A study in Belgium examined the challenges faced by working-class youth, emphasising inequality and discrimination [70]. In Ukraine, research addressed conflicts between personal interests and the common good, highlighting the importance of fostering prosocial behaviour to enhance social cohesion among out-of-school children [71].

A New Zealand study found that unemployment, stigma, ostracism, and social exclusion negatively impact well-being, mental health, and social relationships, affecting both financial stability and sense of belonging [72]. In Greece, young NEETs showed low trust in public institutions and encountered barriers to accessing education and training [73].

In South Africa, where the NEET rate exceeds 30%, studies identified poverty as a major barrier to education and entrepreneurial opportunities [74]. Another study highlighted the importance of stable employment and earnings to address both youth unemployment and poverty [75].

Brazilian research linked teenage pregnancy and school dropout among NEETs to profiles, Such as girls over 15 years old from low-income families who were not generating income [76]. A separate study found that regions with higher social inequality and crime rates had greater proportions of NEET youth [77]. In Buenos Aires, research on underprivileged youth revealed that unequal opportunities based on gender, social sector, and residency status led to a segmented opportunity structure, further perpetuating inequality [78].

#### Childhood adversity

Research has demonstrated the significant impact of childhood adversity on labour market outcomes. A Swedish longitudinal study found that childhood adversities are strongly associated with long-term labour market marginalisation (LMM), including higher risks of unemployment, sickness absence, and disability pensions. The risk of LMM increased with the number of adversities experienced, underlining the long-term consequences of early life stressors [79]. Similarly, a Dutch study showed that adversities such as maltreatment and negative peer influences affect labour market outcomes in young adulthood, with different types of adversity correlating with distinct employment conditions [80].

In Canada, research linked childhood behavioural problems a, such as inattention and aggression, to adverse economic outcomes in adulthood, including unemployment, low income, and financial difficulties [81]. The study suggested that early interventions targeting these behavioural issues could improve education, social integration, and reduce reliance on welfare systems.

### Labour market issues

#### Cyclical variation

Six articles explored the influence of business cycles on young adults, with contributions from the UK, India, the US and Spain. A UK archive study compared how young people were viewed by policymakers during the 1930 s and post 2008, concluding that they were seen as malleable subjects for intervention in both periods [82]. Another UK study found that the relocation propensity of young adults during their traditionally mobile years decreased by 15 percent from 1997 to 2019 [83]. A third UK study found that frequent mental distress among men and women aged 18–34 doubled between 1993 and 2019, aligning trends in mental health and economic recessions [84].

A US study focused on “The Great Recession” (2007-2009) and found that young adults living independently experienced more stress and negative health outcomes from job loss than those living with parents [85]. A Spanish study explored the two last socioeconomic crises in Spain (2008 and 2020), emphasising that the heightened vulnerability of young women and foreign workers in the labour market is a structural issue, not a temporary one [86].

An empirical study from India found that despite high economic growth, youth job losses increased in absolute numbers from 2004-2018, highlighting the severity of employment quality challenges alongside employment quantity [13].

#### Cross-country comparisons

Twelve studies conducted comparative analyses of NEET across countries, with ten focusing on European nations using data from Eurostat, European Social Survey, World Bank databases, and other macro-databases. One study compared the quality of life of NEETs in 24 countries [87], while others examined the impact of the European Social Fund on youth education and employment [88] and assessed how EU member states implemented the EU youth guarantee (YG) recommendation. The latter found that the EU failed to integrate outreach work into member states’ policies, focusing instead on labour market and education efforts [89].

Another EU study identified five clusters of countries based on the factors contributing to high NEET levels [90]. A study on labour market flexibility concluded that policies should incentivise firms to hire young people on permanent contracts to reducse unepmloyment [91]. A comparative study from Southeastern Europe analysed the impact of different educational levels on youth employability, finding a statistically significant negative relationship with NEET rates in most countries [92].

One study examined school-to-work transitions across EU regions, identifying four groups of regions with similar averages and another four with similar trajectories over time [93]. A separate study found that vocationally oriented education systems strengthen the employment – well-being relationship in some countries, where the difference in subjective well-being between employed and unemployed youth is substantial [94].

Two bilateral studies focused on UK and France, as well as England and Portugal. The first concluded that both countries had similar rates of NEET young women and identified barriers such as living in low-income households and limited opportunities [95]. The latter study highlighted the need to move away from an academic education model to better prepare youth for the labour market [96].

A study comparing Canada and the USA found that depressive symptoms during adolescence and early adulthood were linked to low education and NEET status, with Canadians having better education and employment outcomes [97]. Lastly, a study analysed global youth perspectives through Reddit discussions on the “lying flat” movement, noting the limitations of data mainly from English-speaking males in the Global North, with underrepresentation from the Global South [7].

#### Employing NEETs

Research on integrating NEETs into the labour market highlights factors Such as social networks, early work experience, labour market conditions, and the development of psychological or entrepreneurial skills. In Europe, a study across 11 countries found that young men and individuals with higher education were more willing to relocate for employment opportunities, while age reduced this willingness [98]. A UK study showed that individuals with lower qualifications in areas of weak labour demand experienced longer transitions to stable employment [99].

In South Africa, social and family networks played a key role in securing job interviews, often more effectively than formal job applications [100]. Research also emphasized the value of initiatives that develop entrepreneurial skills and psychological resilience, along with holistic employability programmes that combine both soft and hard skill development [101, 102].

In Australia, high-trust environments within industries such as construction helped foster the personal and social capital needed for meaningful employment [103, 104]. In Ghana, structured community education programmes promoted entrepreneurial mindsets as a solution to youth unemployment [105]. However, gender disparities persist, with women in cocoa-growing communities facing higher unemployment rates despite receiving the same training as men [106].

Studies in Sierra Leone and Tanzania showed how resource mobilisation and youth-led initiatives could address unemployment and gender-based violence, empowering youth in agricultural and policy advocacy roles [107, 108]. A multi-country study across Africa, Asia, and the Pacific called for strategies to overcome structural barriers and amplify marginalized voices in policy development [3].

In China, engaging youth in hydroponic farming was shown to require targeted training and pro-environmental education [17], while in India’s Meghalaya region, most unemployed youth aspired to secure any job to sustain their livelihood, with a strong preference for salaried government positions [14]. In Ecuador, women faced significantly higher unemployment rates than men, highlighting ongoing gender disparities [109].

Nordic countries also provide examples of effective policy interventions. Sweden’s 2007 Payroll Tax Reform created 18,100 jobs for young employees through financial incentives [110], and in Norway, early work experience significantly reduced the risk of becoming NEET, particularly among individuals with disabilities or those who left school early [111]. Organisations with a history of hiring NEETs were more likely to do so again, demonstrating the importance of organisational context in creating employment opportunities [112].

### Interventions

Forty-one studies on interventions and services targeting NEETs used diverse methodologies, including qualitative approaches (e.g., in-depth interviews, focus groups, observations, participatory action research, and usability testing) [113–124], and quantitative methods (e.g., surveys, administrative data such as registers, databases, and national records) [47, 125–144]. Some studies employed mixed methods, combining qualitative data from observations and interviews with quantitative data from databases, datasets, and questionnaires [145–152].

Thirteen studies [113, 120, 122, 123, 125, 126, 131–133, 136, 147, 149, 150] provided a broad overview of interventions, covering vocational rehabilitation [122, 125], employment and career support [113, 131–133, 136, 147, 149, 150], and comprehensive personal growth [120, 123, 126].

In a Norwegian study, vocational rehabilitation services tailored to NEETs with mental health conditions emphasised training, placement, and follow-up to support labour market transition, and an American study focused on job search assistance, occupational training, and on-the-job support for better employment outcomes [122, 125]. Early intervention and counselling were recommended to improve programme success [122].

Coordinated career guidance systems [113, 132] and experience-driven career support services [147], which improved psychosocial resources, recognition, and youth employment rate, were found to be critical [113, 132, 147]. Furthermore, innovative career counselling methods were shown to empower marginalised youth by fostering self-worth, adaptability, and career commitment [150].

Research on employment services in Ukraine and The Netherlands emphasised effective communication in employment centres, noting short-term improvements in re-employment but highlighting the need to strengthen public services for sustained outcomes [131, 132]. In Germany, benefit sanctions led to higher transitions to employment, but resulted in lower wages and higher exit rates from the labour force [133]. Turkey’s employment subsidy programme positively impacted employment for young men’s employment [136], while Sweden’s municipal governance efforts reduced NEET rates though collective action [149].

Psychological therapy-focused programmes recommended tailored interventions and improved session attendance, recognising the influence of ethnicity, deprivation, and participation on effectiveness [126]. Canadian and South African studies highlighted individualised support for education, employment, mental health and well-being, with the latter advocating inclusivity and equitable participation through the Needs Ranking approach, adaptable to various populations [120, 123].

Twenty-eight articles examined specific interventions in vocational training [47, 116], employment and career support [117, 121, 127–129, 134, 139–141, 143, 144, 146, 148, 151, 152], and skill development [114, 115, 118, 119, 124, 130, 135, 137, 138, 142, 145].

The two studies highlighting vocational training programmes aimed at enhancing employability through job-related skills and practical training. In Latvia, the Youth Guarantee initiative showed no significant impact on employment outcomes or labour incomes, suggesting the need for programme refinement [47]. In Italy, the “extra-vocational training” programme offered government-funded training for young adults unable to access traditional vocational training. The supportive environment fostered motivation and vocational goal setting, aiding workforce integration, but its overall effectiveness in improving employment outcomes requires further evaluation [116].

Several studies on employment and career support programmes revealed mixed outcomes. In Austria, intensified counselling for disadvantaged unemployed youth failed to improve employment or labour force participation after three years [129]. Greece’s tourism sector training programme, combining classroom-based and on-the-job training, offered limited learning opportunities and career progression [117]. Belgium’s Werkinleving voor Jongeren (WIJ) programme, aimed at enhancing employability through individual guidance, job support, and internships, resulted in lower employment and education rates and higher unemployment compared to standard public employment services, highlighting a need for tailored support for low-education youth [127].

Other programmes showed promise but required additional interventions: In Canada, an online learning platform increased engagement, but did not improve employment outcomes, suggesting the need for hands-on training, in-person engagement, and coaching to enhance online learning impacts. Peer effects emphasised the importance of social interactions in fostering participation [144]. In Spain, a conditional cash transfer programme for youth aged 18-25 failed to increase completion rates for academic years or lower secondary education diplomas, indicating financial initiatives alone were insufficient [134].

Some interventions showed mixed outcomes: In England, traineeships for individuals aged 16-23 aimed to build skills for employment or apprenticeships. While results showed reduced employment among 16-18-year-old, impacts on 19-23-year-old was positive but imprecise [128]. A Hong Kong pilot programme offering career and life development interventions improved career competencies and hope, but stakeholders struggled with implementation challenges [151].

Several promising interventions included Slovakia’s graduate practice intervention, which resulted in participants remaining employed 68 days longer on average than the control group, emphasising the need for rigorous evaluation to optimise policy-making [148]. A Hong Kong entrepreneurship programme improved skills, intentions, and psychological well-being among underprivileged youth, prompting recommendations for early curriculum-based entrepreneurship training in secondary schools [152]. An Australian online platform providing work and study support for young people with mental health challenges achieved significant outcomes and showed potential for cost-effective scalability [146]. Another Australian programme, “Your Job Our Way,” addressed long-term youth unemployment through intensive, tailored support. Success factors included strong client-worker relationships, small caseloads, and social activities to build relational networks and skills [121]. In Ecuador, a six-week employability-support intervention for diverse NEETs, including refugees, significantly reduced psychological distress while improving self-efficacy, self-esteem, and cognitive response inhibition [143].

Three studies from Nigeria, Uganda, and Kenya evaluated agricultural programmes targeting rural youth. Nigeria’s Fadama GUYs programme provided modern agricultural skills and mentorship, empowering 75 % of participants for agribusiness, though barriers like financing, mentorship, and information persisted [139]. The ENABLE-TAAT programme offered six weeks of training, mentorship, and technical assistance, increasing youth incomes by 7 % and improving food security by 75 %, underscoring the potential of agribusiness empowerment initiatives [140]. A rice farming programme in Kenya enhanced formal agricultural training, credit access, and youth organisation memberships, resulting in significant improvements in farm productivity and welfare outcomes. Financial support and training access were identified as key factors influencing participation [141].

Skill development initiatives included Denmark’s NEXT STEP programme, which integrated physical activity into employment interventions, addressing social, physical, and mental barriers to help NEETs transition into education or employment [145]. In Ghana, a sport-based life skills programme improved entrepreneurial competencies, such as goal setting, initiative, cognitive and social skills, highlighting the benefits of such initiatives in youth development [138]. Belgium’s sport employability programme combined mentoring, coaching, and social orientation, but revealed challenges in aligning objectives with participant needs and improving evaluation frameworks [114]. Portugal’s art-based intervention used creative activities, like hip hop, to foster social integration, resilience and community participation among NEETs, emphasised participatory approaches [115]. The Netherlands’ out-of-school programme focused on social-emotional competences (SEL) through diverse themes like arts, politics, philosophy, medicine, and media, with participants reporting long-term SEL benefits [130].

In the UK, flexible alternative education programmes combined physical and mental health initiatives with functional and employment skills, boosting participants’ confidence, well-being, and self-esteem [118]. Moreover, Norway’s game-based positive psychology intervention motivated young adults to pursue education and employment, showing the potential of digital tools to overcome related challenges [119]. Similarly, South Korea’s Kakao NEET Company project demonstrated that online social support and a sense of belonging enhanced well-being, motivation and self-esteem among unemployed youth [124]. Also a Nigerian social-media based intervention effectively taught entrepreneurial skills, such as painting, fashion, and design, positioning social media as a virtual skill acquisition platform [142].

In Hong Kong, a multi-component programme with social workers and trained animals improved socially withdrawn behaviours, employability, and self-esteem among youth, enabling many to transition into work or education [137]. A mindfulness-based intervention in New Zealand reduced distress among young unemployed individuals, suggesting further exploration of its long-term effects [135].

### Consequences of being NEET

While many of the included articles presented above to some degree discuss the consequences of being NEET, seven articles were classified as having consequences as the main theme [45, 153–158]. Four used UK data [153, 156–158], one from Norway [155], one from Turkey [45] and one from multiple European countries [154]. Except for one [154], all used quantitative, longitudinal, methods.

Four studies examined the long-term educational and labour market outcomes of NEET status [153, 155–157]. A British cohort study showed that school absences negatively impacted educational attainment and increased the likelihood of being out of the labour force, highlighting the the need for early intervention to address absenteeism [153]. An analysis of English youth entering the labour market after the 2008-2009 global financial crisis found a strong link between youth unemployment and later mental health issues [158]. Another UK study demonstrated that school exclusions significantly increased the risk of NEET status, unemployment, economic inactivity, and lower earnings by age 25-26, emphasising the need for interventions to prevent exclusions and support reintegration into education and employment [156]. A Scottish study found that NEET status was strongly associated with long-term economic inactivity, with gender disparities, particularly among women, who faced more severe scarring effects. The study underscored the need for gender-specific support [157].

Three studies focused on mental health outcomes. A Spanish study found that early leaving of education and training (ELET) led to decreased self-esteem, depression, and social isolation among youth [154]. A Turkish study found that NEETs had higher rates of mental health issues, obesity, smoking, and alcohol consumption [45].

A Norwegian study of 848,544 individuals born between 1971 and 1986 found significant gender and social origin effects on labour market outcomes. NEET prevalence declined over time, showing gender convergence. However, those with low-educated parents were more likely to face long-term exclusion, highlighting the importance of the timing and duration of NEET status in understanding labour market patterns [155].

### Perspectives on NEET

Studies included in this category provide different perspectives of NEETs, with most being qualitative and involving relatively small sample sizes [159–165].

A South African study examined stakeholder views on strategies to reduce learner dropout in rural secondary schools. It found that a stronger school–community partnership was crucial, with shared responsibility for children’s educational access and opportunities [166]. A French study compared social perceptions of NEETs and found that NEETs were more negatively perceived by others than by themselves [167].

In interviews with 256 teachers from Italy, Lithuania, Portugal, and Romania, one study concluded that psychosocial competencies among teachers are essential for effectively managing early school-leaving situations [168].

### Other topics

Six studies were classified under the “Other topics” category [169–174]. Five of them had a qualitative approach [169, 170, 172–174], and one was quantitative [171]. The studies came from Norway [169, 174], the UK [171], Central and South Europe [172, 173], and the US [170].

Several studies focused on youth development, employment, and social interactions. Three examined personal development and interaction [169, 170, 173]. A Norwegian study found that waiting for services is a form of social interaction, influenced by power dynamics and agency, with staff aiming to reduce waiting times to enhance participant well-being [169]. A US study explored youth camp experiences for low-income youth, finding that 33 % of written narratives showed growth in learning, perspective shifts, resilience, and passion discovery [170]. In Spain, out-of-school programmes focused on building trust and support in youth-adult partnership [173].

Three studies addressed policy and strategy [171, 172, 174]. A UK study found significant variations in NEET policies, highlighting the need for a cohesive UK-wide strategy and raising concerns about sustainability due to funding limitations and lack of evidence on effective interventions [171]. A study on Public Employment Services (PES) in Southern Mediterranean countries emphasized the importance of enhancing relationships and building strategic action in rural areas to support NEETs [172]. A Norwegian study revealed that municipalities with lower NEET rates had more integrated services, with effective communication and collaboration being key to addressing NEETs’ complex needs [174].

## Discussion

This global scoping review synthesised a diverse and evolving body of literature on NEET-individuals, with a focus on understanding their challenges, the consequences of disengagement, and the effectiveness of different types of interventions. While the heterogeneity of NEET populations and the variability across regions present analytical challenges, key patterns emerge regarding barriers, long-term outcomes, and promising strategies for support. In this discussion, we reflect on these findings and highlight implications for both practice and future research.

### Consequences of NEET

Understanding the trajectories of individuals who have experienced being NEET provides critical insights into long-term patterns of social and economic exclusion, informing more tailored and effective interventions. Half of the six included studies that primarily examined the long-term educational and labour market outcomes of NEET status underscored the importance of addressing early school absenteeism. These studies emphasized the detrimental impact of school exclusion on future labour market prospects, highlighting the need for targeted support to reintegrate excluded students [153, 156, 158]. Additionally, NEETs face significant health risks, including substance abuse and mental health issues, and tend to report lower levels of well-being and future orientation. Successful transitions to employment can notably improve subjective well-being, though with distinct gender differences [155]. Studies from both Europe and the US indicate that women, despite surpassing men in educational attainment, face greater challenges in the labour market, including higher inactivity and a persistent wage gap [25–27]. Men, on the other hand, exhibit greater sensitivity to labour market changes, with their life satisfaction being more heavily influenced by employment status. Unemployment disproportionately impacts men’s happiness, reflecting traditional gender roles and income strategies [175]. Educational experiences also show gendered patterns, where women initially report lower life satisfaction but see improvements during the transition to work, while men experience a sharper decline, followed by gradual recovery.

In Norway, educational, social, and health challenges in early life have been found to significantly affect participation in work, with women showing a stronger educational gradient [32]. This underscores the importance of addressing these early life factors to improve long-term work outcomes. Research from southern parts of Europe further highlights gender disparities in NEET status, with women facing higher risks, especially as they age, and the mixed effectiveness of initiatives like the Youth Guarantee in engaging vulnerable groups [34–36]. Policymakers should focus on both mental health and gender disparities in their interventions to improve labour market inclusion and well-being outcomes for NEETs [154, 155, 157, 158].

### Barriers faced by NEETs

NEETs encounter a range of psychological and socioeconomic barriers that hinder their ability to engage in education, employment, or training. Many studies highlight the importance of early intervention for youth showing signs of mental health issues, as addressing these problems before they escalate can prevent educational and work-related marginalization. Integrated approaches that combine mental health services with educational and vocational training are essential to reduce the risk of NEET status. For instance, providing mental health support during critical transitions, such as the move from school to work, can significantly improve long-term educational and employment outcomes. Specific groups, including those with personality disorders or neurodevelopmental conditions, require tailored interventions, such as counselling, mentoring, and workplace accommodations, to help them remain engaged in productive activities.

Moreover, the characteristics of NEETs reveal additional barriers rooted in self-resources, non-cognitive skills, and socio-economic factors. Low self-esteem, self-efficacy, and lack of employment hope are critical factors that negatively impact the psychological well-being of unemployed young adults. Strengthening these self-resources through targeted interventions can mitigate mental health issues and improve employability [23]. Similarly, research has found that NEET status erodes non-cognitive skills, such as conscientiousness, extraversion and grit, further compounding the socio-economic challenges faced by these individuals [24]. Addressing these psychological and socio-economic barriers through holistic approaches is crucial for improving the well-being and prospects of NEET youth.

### What works for NEETs?

Studies exploring interventions and services targeting NEETs highlight several key factors that contribute to successful outcomes. Tailored interventions that address the diverse and complex needs of NEETs are particularly effective, with individualised career development plans and holistic approaches that integrate physical, mental, and social support proving essential. These interventions should not only focus on immediate employment but also on sustained support to ensure long-term impact, as several programmes showed a decline in outcomes over time. Thus, ongoing follow-up mechanisms and well-defined outcomes are necessary to track progress and assess effectiveness. Moreover, involving NEETs in the design and implementation of interventions has been shown to enhance their relevance and impact. By engaging participants in decision-making processes, interventions become more meaningful and better aligned with their actual needs.

Implementing programmes that build social networks and provide intermediary support can further assist NEETs in securing employment opportunities [104], while fostering trust and support in youth-adult partnerships enhances personal growth and community engagement, creating environments for meaningful social interactions [173]. Additionally, enhancing collaboration among service providers can lead to more integrated services for NEETs, fostering partnerships between public employment services, education institutions, and social services [174]. To support these approaches at scale, policymakers should incentivise the development of integrated youth services that combine vocational training, mental health support, and social skill-building. National and local governments should also promote formal collaboration between education, health, and labour sectors to ensure continuity and accessibility across support systems.

Innovative approaches, such as digital platforms and game-based interventions, have demonstrated promising potential for engaging and supporting NEETs. These technological solutions offer scalable and cost-effective ways to provide continuous support and skill development. Furthermore, studies also suggest the value of involving young people in the research process itself. Peer research and participatory methodologies [176], allow NEETs to take an active role in the creation of solutions, fostering empowerment and promoting social change [78]. Research projects that employ co-creation and human-centred design processes ensure that interventions are developed with direct input from the young people they aim to help, leading to more tailored and effective programmes [115, 119, 123, 145].

In terms of skill development, there has been a shift in the literature toward recognising that many NEETs are far from the labour market, and that progress should be measured in steps rather than immediate employment. Comprehensive personal growth programmes, which emphasize gradual development towards education or employment, have been found to be effective. These programmes acknowledge the complex barriers faced by NEETs and support them sustainably, fostering their development through incremental milestones. In addition, governments should adopt policies that recognise and fund stepwise progress models for NEETs who are far from the labour market. Rather than focusing solely on employment outcomes, funding frameworks should reward intermediate milestones related to personal development, training engagement, and mental well-being. By integrating personal growth, tailored support, and active engagement, these approaches provide NEETs with the tools and confidence needed to achieve long-term educational and employment goals.

### Gaps in the literature and future research

Despite the growing body of research on NEETs, several significant gaps remain [3]. One key criticism is that the NEET label itself is too broad and overly heterogeneous, grouping together individuals with vastly different experiences, characteristics, and needs [177, 178]. This lack of disaggregation makes it difficult to design targeted interventions, as the challenges and support needs of a long-term unemployed graduate may differ substantially from those of a young carer or a person with a disability. Future research should prioritise identifying and analysing NEET subgroups to improve the precision and impact of policy responses.

In addition, there is limited engagement with the subjective experiences of young people themselves. Few studies explore how NEETs perceive and navigate their situations, leading to interventions that may not fully reflect their needs or aspirations. Greater inclusion of youth perspectives — through participatory and co-creative approaches — could enhance the relevance and effectiveness of future programmes.

The literature also tends to have a geographical bias, with much of the research concentrated in certain regions, particularly high-income countries. This leaves gaps in understanding the challenges faced by NEETs in other contexts, particularly in low- and middle-income countries, where barriers to education and employment may differ significantly and where structural barriers may be more pronounced [165]. A more globally representative research base is needed to support inclusive policy development.

Another overlooked area is the role of domestic, voluntary, and other forms of non-remunerated work. Many individuals categorised as NEET engage in caregiving, informal labour, or community-based activities that build valuable skills and contribute to society. However, these contributions are often excluded from formal definitions of work and education, leading to an incomplete picture of youth activity and potential. Future studies should examine how such informal roles shape identity, skill development, and future labour market trajectories.

While digital interventions have shown promise in supporting NEETs’ transitions into education and employment, their long-term effectiveness remains under-researched. Studies have explored innovative approaches such as online learning platforms, digital mental health support, and gaming-based interventions [119, 124, 142, 144, 146]. However, many of these programmes lack integration with broader psychosocial support systems, and their sustainability beyond short-term outcomes is unclear. Further longitudinal research is needed to assess how digital approaches can be designed to support lasting educational and employment outcomes, particularly when combined with mentoring, peer networks, or hybrid service models.

Expanding opportunities for social participation and emotional support, particularly through technology, could address key gaps in current interventions and more effectively address the complex barriers faced by NEET populations. Moving forward, addressing NEET heterogeneity, recognising informal contributions, and strengthening the evidence base on digital interventions will be essential to developing more equitable and effective support systems.

Although this review did not include a formal quality appraisal, a general observation across themes is the predominance of cross-sectional, descriptive, and survey-based designs. While these approaches provide valuable insights into the characteristics and distribution of NEET status, they are often limited in their ability to capture change over time or to infer causal relationships, particularly in the context of evaluating interventions. Fewer studies employed longitudinal or mixed-methods designs, and those that did were typically concentrated in high-income countries. This methodological imbalance highlights the need for a broader range of robust research designs, particularly in underrepresented regions and among NEET subgroups, to strengthen the evidence base and better inform policy and practice.

### Strengths and limitations of the scoping review

This scoping review presents a comprehensive mapping of the global literature surrounding NEETs, with a notable strength being its inclusion of research from diverse geographical contexts, including low-income and non-Western countries [165]. This breadth is often lacking in existing literature, and by encouraging future NEET-researchers to incorporate a wide range of studies and treat them equally in analyses, we hope to foster a more global understanding of the NEET phenomenon. Despite significant differences in labour markets and socio-economic frameworks across regions, the universal implications of NEET status highlight the relevance of this issue worldwide.

One inherent limitation of scoping reviews is the lack of critical evaluation of the studies included, which prevents us from identifying gaps in the literature based on quality and bias. The body of literature on NEETs encompasses a variety of methodologies, including a significant amount of qualitative and mixed-methods studies, making quality assessment challenging. Thus, the scoping review methodology [19] was deemed appropriate for our aim of informing the development of policies and practices to better support NEET individuals.

Additionally, while efforts were made to mitigate potential oversights, there is a possibility that some relevant studies were missed due to our selection of databases and the time constraints of our search (covering publications from 2021 to April 2024). We attempted to counterbalance this by systematically reviewing references in existing literature.

We also excluded studies related to serious health conditions and pandemic-specific research, recognising that these decisions might limit the comprehensiveness of our findings. Furthermore, our focus on English literature means that we may have overlooked valuable evidence presented in other languages. This language restriction may have introduced bias and led to the exclusion of relevant findings reported in other languages, particularly from regions underrepresented in international publishing.

Finally, this review was conducted without applying a predefined theoretical framework, in line with its exploratory purpose. While this allowed for a broad and inclusive mapping of the literature, it may limit the conceptual integration of findings across levels. Future reviews may benefit from situating the evidence within a formal theoretical model to support hypothesis generation or intervention design [4].

Acknowledging these limitations is essential for contextualising our findings and guiding future research in this area.

## Conclusions

This scoping review provided a comprehensive global mapping of research on NEET-individuals. By synthesizing studies from a wide range of geographical, methodological, and thematic perspectives, the review contributes to a deeper understanding of the NEET phenomenon, its complexity, and the evolving strategies used to address it. While existing evidence underscores the importance of integrated, youth-centered, and context-sensitive solutions, substantial gaps remain—particularly with regard to subgroup-specific needs, youth voice, and long-term impact evaluation.

To advance the field and improve support for NEET individuals, we suggest three key priorities for future research and practice; i) Disaggregate NEET subgroups to better reflect the diverse pathways, risks, and support needs within this population—including gender, health status, and socio-economic background; ii) Evaluate digital and hybrid interventions longitudinally, with attention to sustainability, equity, and the integration of mental health and psychosocial support; iii) Embed youth voices more systematically in the design, implementation, and evaluation of interventions, using participatory and co-creative methods that ensure relevance and empowerment.

Pursuing these priorities will help ensure that NEET-targeted policies and programmes are inclusive, evidence-informed, and responsive to the lived realities of young people in varied global contexts.

## Supplementary Information

Supplementary Material 1.

Supplementary Material 2.

## Data Availability

All data generated or analysed during this study are included in this published article and its supplementary information files.

## Abbreviations

ADHD: Attention-deficit/Hyperactivity disorder
AI: Artificial intelligence
ELET: Early leaving of Education and training
EU: European Union
IPS: Individual placement and support
JBI: Joanna Briggs Institute
LMM: Labour market marginalization
NEET: Not in Education, Employment, or Training
PCC: Population-concept-context
PES: Public employment services
PRISMA: Preferred reporting Items for systematic reviews and meta-analyses
SEL: Social-emotional learning
UK: United Kingdom
YG: Youth guarantee
