# Supplementary material for: Young adults not in education, employment, or training (NEET): a global scoping review
Source: BMC Public Health. 2025 Oct 8;25:3394. doi: 10.1186/s12889-025-24781-y (PMC12505988; doi:10.1186/s12889-025-24781-y)
Supplement: Supplementary file 1 — Supplementary Material 1. [file 12889_2025_24781_MOESM1_ESM.docx]

**PubMed. Date of search: 10.04.2024**

| # | Search | Results |
| --- | --- | --- |
| #1 | NEET[Title/Abstract] OR unemploy*[Title/Abstract] OR "out of work"[Title/Abstract] OR "out of school"[Title/Abstract] OR "socially excluded"[Title/Abstract] OR "not in work"[Title/Abstract] OR "not in employment"[Title/Abstract] OR "not in education"[Title/Abstract] OR precarious[Title/Abstract] OR "without income"[Title/Abstract] OR "job seeking"[Title/Abstract] OR "job loss"[Title/Abstract] | 32,122 |
| #2 | adolescen*[Title/Abstract] OR teen*[Title/Abstract] OR youth*[Title/Abstract] OR young*[Title/Abstract] OR student*[Title/Abstract] OR minors[Title/Abstract] OR juvenile[Title/Abstract] OR pupil*[Title/Abstract] | 1,663,197 |
| #3 | "return to work"[Title/Abstract] OR "occupational rehabilitation"[Title/Abstract] OR "work rehabilitation"[Title/Abstract] OR "vocational treatment"[Title/Abstract] OR "vocational rehabilitation"[Title/Abstract] OR recreation[Title/Abstract] OR "evidence-based practice"[Title/Abstract] OR reintegration[Title/Abstract] OR peer*[Title/Abstract] OR "one-to-one support"[Title/Abstract] OR group*[Title/Abstract] OR advisor*[Title/Abstract] OR counsel*[Title/Abstract] OR mentor*[Title/Abstract] OR "motivational interview"[Title/Abstract] OR "psychological approach"[Title/Abstract] OR mental[Title/Abstract] OR therapy[Title/Abstract] OR guidance[Title/Abstract] OR "self-esteem building"[Title/Abstract] OR "personality development"[Title/Abstract] OR "volunteer* program*"[Title/Abstract] OR "learning program*"[Title/Abstract] OR outdoor*[Title/Abstract] OR hiking[Title/Abstract] OR sport[Title/Abstract] OR nature[Title/Abstract] OR pet[Title/Abstract] OR animal*[Title/Abstract] OR physiotherapy[Title/Abstract] OR "physical activity"[Title/Abstract] OR "physical fitness"[Title/Abstract] OR exercise[Title/Abstract] OR nutrition[Title/Abstract] OR diet[Title/Abstract] OR art[Title/Abstract] OR dance[Title/Abstract] OR music[Title/Abstract] OR relaxation[Title/Abstract] OR "pain management"[Title/Abstract] OR "learning difficulties"[Title/Abstract] OR dyslexia[Title/Abstract] OR "learning disability"[Title/Abstract] OR yoga[Title/Abstract] OR mindful*[Title/Abstract] OR lifestyle[Title/Abstract] OR habit*[Title/Abstract] OR "circadian rhythm"[Title/Abstract] OR sleep[Title/Abstract] | 10,000,429 |
| #4 | covid"[Title/Abstract] OR "pandemic"[Title/Abstract] OR "severe mental*"[Title/Abstract] OR "serious mental*"[Title/Abstract] OR "stroke"[Title/Abstract] OR "psychosis"[Title/Abstract] OR "cancer"[Title/Abstract] OR "injury"[Title/Abstract] OR "severe autism"[Title/Abstract] OR "HIV"[Title/Abstract] OR "congenital heart*"[Title/Abstract] OR "diabetes"[Title/Abstract] OR "sickle cell anemia"[Title/Abstract] OR "chronic disease"[Title/Abstract] OR "schizophrenia"[Title/Abstract] OR "handicap"[Title/Abstract] OR "severe neurologic*"[Title/Abstract] OR "intellectually disable*"[Title/Abstract] OR "multiple sclerosis"[Title/Abstract] OR "suicid*"[Title/Abstract] OR "substance"[Title/Abstract] OR "IPS"[Title/Abstract] OR "individual placement and support"[Title/Abstract] | 5,328,881 |
| #5 | #1 AND #2 AND #3 NOT #4  Filter:   - Publication year: 2021, 2022, 2023, 2024 - Language: English | 642 |

**Scopus. Date of search: 11.04.2024**

| # | Search | Results |
| --- | --- | --- |
| #1 | TITLE-ABS-KEY ( neet OR unemploy* OR "out of work" OR "out of school" OR "socially excluded" OR "not in work" OR "not in employment" OR "not in education" OR precarious OR "without income" OR "job seeking" OR "job loss" ) | 124,300 |
| #2 | TITLE-ABS-KEY (adolescen* OR teen* OR youth* OR young* OR student* OR minors OR juvenile OR pupil*) | 6,666,734 |
| #3 | TITLE-ABS-KEY ( "return to work" OR "occupational rehabilitation" OR "work rehabilitation" OR "vocational treatment" OR "vocational rehabilitation" OR recreation OR "evidence-based practice" OR reintegration OR peer* OR "one-to-one support" OR group* OR advisor* OR counsel* OR mentor* OR "motivational interview" OR "psychological approach" OR mental OR therapy OR guidance OR "self-esteem building" OR "personality development" OR "volunteer* program*" OR "learning program*" OR outdoor* OR hiking OR sport OR nature OR pet OR animal* OR physiotherapy OR "physical activity" OR "physical fitness" OR exercise OR nutrition OR diet OR art OR dance OR music OR relaxation OR "pain management" OR "learning difficulties" OR dyslexia OR "learning disability" OR yoga OR mindful* OR lifestyle OR habit* OR circadian AND rhythm OR sleep ) | 431,473 |
| #4 | TITLE-ABS-KEY ( ( covid OR pandemic OR "severe mental*" OR "serious mental*" OR stroke OR psychosis OR cancer OR injury OR "severe autism" OR hiv OR "congenital heart*" OR diabetes OR "sickle cell anemia" OR "chronic disease" OR schizophrenia OR handicap OR "severe neurologic*" OR "intellectual* disable*" OR "multiple sclerosis" OR suicidal* OR substance OR ips OR "individual placement and support" ) ) | 10,185,396 |
| #5 | #1 AND #2 AND #3 AND NOT #4  Filter:   - Publication year: 2021, 2022, 2023, 2024 - Language: English | 43 |

**Web of science – topics (TS)**^[[1]](#footnote-1)^**. Date of search: 10.04.2024**

| # | Search | Results |
| --- | --- | --- |
| #1 | TS=(NEET OR unemploy* OR "out of work" OR "out of school" OR "socially excluded" OR "not in work" OR "not in employment" OR "not in education" OR precarious OR "without income" OR "job seeking" OR "job loss") (All Fields) | 83,154 |
| #2 | TS=(adolescen* OR teen* OR youth* OR young* OR student* OR minors OR juvenile OR pupil*) | 3,199,505 |
| #3 | TS=("return to work" OR "occupational rehabilitation" OR "work rehabilitation" OR "vocational treatment" OR "vocational rehabilitation" OR recreation OR "evidence-based practice" OR reintegration OR peer* OR "one-to-one support" OR group* OR advisor* OR counsel* OR mentor* OR "motivational interview" OR "psychological approach" OR mental OR therapy OR guidance OR "self-esteem building" OR "personality development" OR "volunteer* program*" OR "learning program*" OR outdoor* OR hiking OR sport OR nature OR pet OR animal* OR physiotherapy OR "physical activity" OR "physical fitness" OR exercise OR nutrition OR diet OR art OR dance OR music OR relaxation OR "pain management" OR "learning difficulties" OR dyslexia OR "learning disability" OR yoga OR mindful* OR lifestyle OR habit* OR "circadian rhythm" OR sleep) | 14,722,223 |
| #4 | TS=(covid OR pandemic OR "severe mental*" OR "serious mental*" OR stroke OR psychosis OR cancer OR injury OR "severe autism" OR HIV OR "congenital heart*" OR diabetes OR "sickle cell anemia" OR "chronic disease" OR schizophrenia OR handicap OR "severe neurologic*" OR "intellectual* disable*" OR "multiple sclerosis" OR suicid* OR substance OR IPS OR "individual placement and support") | 7,245,106 |
| #5 | #1 AND #2 AND #3 NOT #4  Filter:   - Publication year: 2021, 2022, 2023, 2024 - Language: English | 1,638 |

1. A search by Topic will search in the title, abstract and keyword fields of Web of Science records. [↑](#footnote-ref-1)
